# Measuring body temperature in birds – the effects of sensor type and placement on estimated temperature and metabolic rate

**DOI:** 10.1242/jeb.246321

**Published:** 2023-12-12

**Authors:** Fredrik Andreasson, Elin Rostedt, Andreas Nord

**Affiliations:** Department of Biology, Section for Evolutionary Ecology, Lund University, Ecology Building, SE-223 62 Lund, Sweden

**Keywords:** Heterothermy, Thermoregulation, Endotherm, PIT tag, *Parus major*, RFID

## Abstract

Several methods are routinely used to measure avian body temperature, but different methods vary in invasiveness. This may cause stress-induced increases in temperature and/or metabolic rate and, hence, overestimation of both parameters. Choosing an adequate temperature measurement method is therefore key to accurately characterizing an animal's thermal and metabolic phenotype. Using great tits (*Parus major*) and four common methods with different levels of invasiveness (intraperitoneal, cloacal, subcutaneous, cutaneous), we evaluated the preciseness of body temperature measurements and effects on resting metabolic rate (RMR) over a 40°C range of ambient temperatures. None of the methods caused overestimation or underestimation of RMR compared with un-instrumented birds, and body or skin temperature estimates did not differ between methods in thermoneutrality. However, skin temperature was lower compared with all other methods below thermoneutrality. These results provide empirical guidance for future research that aims to measure body temperature and metabolic rate in small bird models.

## Teaser

**Summary:** Comparison of four common body temperature measurement methods shows that none of them affect metabolic rate compared with controls in great tits.

## INTRODUCTION

Homeothermic endotherms maintain a high and relatively constant body temperature independent of air temperature by physiological regulation of heat production and dissipation. However, body temperature varies on daily and seasonal scales in response to, for example, changes in resource availability or ambient conditions (Hainsworth et al., 1977; Powers et al., 2003; Nowack et al., 2015; Barak et al., 2018; Romano et al., 2019), physiological (e.g. Guillemette and Pelletier, 2022) and immunological state (Kluger, 1991), or as a normal part of the diurnal cycle (Aschoff and Pohl, 1969).

Technological advancements have made it easier to measure body temperature continuously and a range of options with varying levels of invasiveness are now available, including internally implanted or ingested devices [loggers, radio-transmitters or passive integrated transponder (PIT) tags], peripherally attached instruments (e.g. radio-transmitters, PIT tags, thermistors or thermocouples), and non-contact methods such as infrared thermography (for a review of these options, see McCafferty et al., 2015). All methods that are based on implantation, ingestion or attachment require (sometimes repeated) capture and handling that can be difficult to execute under field conditions, and the devices themselves will also differ in the level of disturbance caused to the animal. Any procedure that causes stress or discomfort may be associated with thermoregulatory responses, most often observed as stress-induced hyperthermia (SIH) that is characterized by elevated core body temperature (Cabanac and Briese, 1991; Cabanac and Guillemette, 2001; Nord and Folkow, 2019) and sometimes also reduced skin temperature (e.g. Herborn et al., 2015; Jerem et al., 2015; Nord and Folkow, 2019), due to a combination of increased metabolic heat production and peripheral vasoconstriction (Oka and Oka, 2012). Use of stressful methods therefore risk distorting the purpose of the measurement by, depending on sensor placement, overestimating or underestimating body temperature. As both acute and chronic stressors have been shown to increase energy expenditure (Kuti et al., 2022), the use of a stressful measurement method may also cause an overestimation of metabolic rate if the two traits are measured simultaneously to characterize the thermoregulatory phenotype. The extent to which this risk is realized has not, to the best of our knowledge, been subject to any empirical test.

Tissue temperature also varies depending on where, along the thermal gradient from core to periphery, it is measured because of both regulatory processes (i.e. local heterothermy, e.g. Barnes, 1989; Eichhorn et al., 2011; Rummel et al., 2019) and heat flux properties. Thus, gaining insight into the temporal patterns of body temperature requires continuous and careful measurement that considers the most appropriate measurement location and method, weighing the benefits of reduced severity against measurement accuracy. Several recent avian studies have measured body temperature and metabolic rate simultaneously to evaluate thermal and metabolic responses to hot (e.g. Smith et al., 2017; Pollock et al., 2021; Freeman et al., 2022) and cold (e.g. Nord and Folkow, 2018; Andreasson et al., 2020a) conditions but, to the best of our knowledge, there are no empirical studies that compare directly how well a given measurement technique functions across environmental conditions and the extent to which each method is associated with stress-related alterations to either temperature or metabolism.

Using wild-caught great tits (*Parus major*), we assessed how four common methods that differ in perceived invasiveness and site of measurement – intraperitoneal, cloacal, subcutaneous and cutaneous – performed in ambient temperatures in thermoneutrality (25°C), close to the lower critical temperature (5°C) and far below thermoneutrality (−15°C), and how these methods affected simultaneous measurements of resting metabolic rate (RMR). Intraperitoneal measurements have been used extensively, in both arid and hot climates (recent examples include Whitfield et al., 2015; Albright et al., 2017; McKechnie et al., 2017; Smith et al., 2017; Oswald et al., 2018; Freeman et al., 2022) and in colder environments (Reinertsen, 1982; Reinertsen and Haftorn, 1986; Schaeffer et al., 2015; Appenroth et al., 2021), and are widely considered as non-stressful and accurate when the animals have recovered from surgery. Cloacal, rectal or colonic temperature is usually recorded as a single-point measurement but has also been recorded continuously (see Thomas and Maclean, 1981; Nord and Folkow, 2018; Andreasson et al., 2020b; Wolf et al., 2020; Pollock et al., 2021). These measurements, though presumably providing accurate information on core temperature (at least in small-bodied species), have also been reported to cause stress (McKechnie et al., 2016) and SIH (Bouwknecht et al., 2007). Subcutaneous implants have been used widely over the last decade or so (Wacker et al., 2012; Nord et al., 2013; Sköld-Chiriac et al., 2015; Oswald et al., 2018; Andreasson et al., 2020a,c; Nilsson et al., 2020; Tapper et al., 2020a,b, 2021, 2022) and are typically perceived as minimally invasive, but will not always reflect core temperature unless calibrated (Nord et al., 2013; Sköld-Chiriac et al., 2015; but see Oswald et al., 2018). Other studies have measured skin temperature by cutaneous thermistors or thermocouples (Hiebert, 1991; Bech et al., 1997; Nord et al., 2016, 2020; Nord and Folkow, 2018, 2019) – measurements that may be more affected by ambient conditions because of the peripheral location of the sensor (e.g. Nord et al., 2016; Adelman et al., 2010), or be associated with stress if the animal is tethered to a thermocouple. Based on previous studies and our perception of the invasiveness of the four methods, we predicted that birds with externally attached thermocouples would have a higher metabolic rate compared with those with implanted tags (and controls). We also predicted that body temperature measurements would decrease along the gradient from the body core (intraperitoneal and cloacal) to periphery (subcutaneous and cutaneous) and that any difference in body temperature between the internal and peripheral measurements would be most pronounced at low ambient temperature as peripheral sensors are likely to be disproportionally affected by low temperatures as a result of their distal placement and/or by cold-induced peripheral vasoconstriction (Robertson et al., 2020).

## MATERIALS AND METHODS

We studied great tits (*Parus major* Linnaeus), a 15–20 g passerine, and a model species for studying metabolic rate and body temperature in ecology and evolution (e.g. Haftorn, 1972; Reinertsen and Haftorn, 1986; Røskaft et al., 1986; Carere and van Oers, 2004; Broggi et al., 2004, 2007; Caro and Visser, 2009; Amo et al., 2011; Bouwhuis et al., 2011; Mathot et al., 2016; Ruuskanen et al., 2016; Verhagen et al., 2019; Nilsson et al., 2020) from a nest-box population close to the village of Vomb in southernmost Sweden (55°39′N, 13°33′E). The area, dominated by planted pines *Pinus sylvestris* but with a mixed, deciduous understory contains ca. 400 nest boxes. Great tits in this population routinely use nest boxes as roosting sites in winter. Between 25 January and 18 February 2022, starting at sunset, we caught roosting great tits and brought them to the animal housing facilities at Lund University, where they were housed in individual cages (43×71×58 cm) at 5°C and subjected to a simulated natural photoperiod. Two of the individuals were not collected in Vomb, but from a nest-box population close to Lund University (Furulund, 55°46′23.1918″N, 13°4′21.8994″E). Mean (±s.d.) daily temperature in Lund from 25 January to 18 February in 2017–2021 was 1.3±3.7°C (Swedish Meteorological and Hydrological Institute, 2023; https://www.smhi.se/data/meteorologi/temperatur, station number 53430 - Lund). We provided the birds with water, tallow, and a mix of sunflower seeds and granulated peanuts *ad libitum*, as well as 5–10 mealworms daily.

All birds were ringed and measured (maximum wing chord ±0.5 mm, tarsus length ±0.1 mm, body mass ±0.1 g), and aged (birds in their second calendar year: 2cy, birds in their third calendar year or older: 3cy+) and sexed based on plumage characteristics (Svensson, 1992). We assigned the birds to an experimental category for body temperature measurements (see below), which were completed 24–72 h after capture. We assigned birds to measurement methods with the objective to obtain a balanced design with regards to age and sex of the birds (see Table S1 for sample sizes and biometrics) and with the aim of including 13–15 birds in each category, which we deemed sufficient to detect biologically relevant differences based on previous experience with the model species. All birds were released at the capture site by the end of the measurements. Ethical approval was granted by the Malmö/Lund Research Animal Committee (no. 9426-19). Catching and ringing of birds was performed with permission from the Swedish Ringing Centre (licence no. 475).

### Body temperature measurement methods

**Fig. 1.**
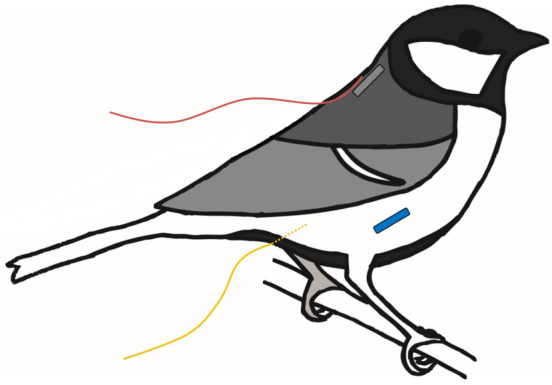
**A schematic figure of a great tit (*Parus major*) illustrating placement and sensor type for the four methods used for body temperature measurements.** Core body temperature was measured using either an intraperitoneally implanted PIT tag (IP – blue) or a thermocouple inserted into the cloaca (CL – yellow). Peripheral body temperature was measured using either a subcutaneously implanted PIT tag (SC – grey) or a thermocouple glued to the skin surface (CU – red). Each individual bird was measured with only one type of sensor.

We measured body temperature using four different methods (see Fig. 1 for a schematic representation of the measurement locations, one method per individual), of which two were assumed to represent peripheral temperature and two core body temperature: intraperitoneal (IP; core temperature); cloacal (CL; proxy for core temperature); subcutaneous (SC; peripheral temperature) and cutaneous (CU; peripheral temperature). IP birds were locally anaesthetized with lidocaine and prilocaine (EMLA, APP Pharmaceuticals, Lake Zurich, IL, USA) 15 min before implantation. Then, we sterilized the skin surrounding the distal tip of the sternal keel using 70% ethanol and used a 12-gauge syringe to pierce the skin. The incision was widened using a 15C scalpel blade, after which a sterile temperature-sensitive PIT tag (BioTherm13-Biomark, Boise, ID, USA; height: 13.0 mm; diameter: 2.1 mm) was inserted. The incision was sealed using cyanoacrylate adhesive (UHU Super Glue, Bühl, Germany) and covered with antiseptic ointment (1% H_2_O_2_: LHP, Bioglan, Malmö, Sweden). SC birds were implanted dorsally on the neck, immediately lateral to the spine, using the same type of PIT tag but without applying anaesthesia prior to sterilization and implantation.

Body temperature in the CL and CU birds was measured using 26-gauge type T (copper–constantan) thermocouples. Skin temperature (CU) was measured by attaching a thermocouple directly onto the skin, dorsally on the neck such that the temperature-sensitive junction was positioned to match that of the subcutaneous implant in the SC birds. To increase the area of adhesion, a square piece of surgical tape (10×5 mm) was placed immediately below the tip of the thermocouple wire so that the wire could be glued to the skin without any risk of covering the thermosensitive junction with adhesive. Cloacal temperature (CL) was measured by securing a thermocouple wire 12 mm into the cloaca. This was achieved by gluing (using cyanoacrylate adhesive) two pieces of 20×5 mm surgical tape attached to the wire onto the tail feathers. Previous work has shown that further insertion does not alter the temperature reading in similarly sized models (e.g. Nord et al., 2009). The thermocouples were attached immediately before measurements of metabolic rate and body temperature, and were removed at the end of the experiment. We also included an unmanipulated control group (C) in the study to obtain baseline data, undisturbed by surgery or thermocouple attachment, of metabolic rate.

### Measurement of metabolic rate and body temperature

RMR was calculated based on measurements of oxygen consumption and carbon dioxide production using flow-through respirometry within the thermoneutral zone at 25°C, close to the lower critical temperature (12.9°C; A.N., unpublished data) at 5°C, and far below thermoneutrality at −15°C. All measurements started after sunset (mean±s.d. local start time: 18:10±28 min) and ended at, or before, sunrise (mean±s.d. local end time: 07:03±2 min). The birds were put individually in 1 litre hermetic glass chambers placed inside a dark, temperature-controlled climate test chamber (Weiss Technik WK3 C180, Reiskirchen, Germany). The chambers were ventilated using dry (Drierite, Hammond Drierite Company, Xenia, OH, USA), pressurized air at a flow rate of 594±22 ml min^−1^ (mean±s.d., standard temperature and pressure, dry, STPD: measured using an FB-8 mass flow meter, Sable Systems International, Las Vegas, NV, USA). We sub-sampled excurrent 151±9 ml min^−1^ (mean±s.d.) air from the main air stream using a SS4 sub-sampler (Sable Systems International). Once water vapour density (using an RH-300 humidity meter, Sable Systems International) and carbon dioxide (using a CA-10 carbon dioxide meter, Sable Systems International) had been measured, we scrubbed water vapour and CO_2_ from the air stream using a combination of Drierite and Ascarite(II) (Arthur H. Thomas Company, Philadelphia, PA, USA) and then analysed the gas stream for oxygen (FC-10A, Sable Systems International). The O_2_ meter was span calibrated to 20.95% using dry room air. The CO_2_ meter was zero calibrated using 100% N_2_ and span-calibrated using 0.5% CO_2_ in N_2_. The humidity meter was zero calibrated using dry room air and was span calibrated by solving for water vapour pressure based on changes in O_2_ concentration when measuring dry (Drierite) and moist room air, using Dalton's law of partial pressure (Lighton, 2019).

Body temperature was measured every second, in the CL and CU groups by a TC-2000 thermocouple box (Sable Systems International) and in the SC and IP groups by a custom-made receiving system (Biomark, Boise, ID, USA) with antennae placed directly below the respirometry chambers.

We measured 5 birds sequentially during a night. Each measurement cycle (80 min) started and ended with a 15 min baseline, in between which birds were sampled sequentially for 10 min each. The air temperature in the climate chamber changed every 160 min (i.e. after two full measurement cycles) to generate a temperature profile of 25, 5, −15, 5 and 25°C (only one cycle at 25°C in the morning) during the night (Fig. S1).

O_2_ consumption rate (*V̇*_O_2__) and CO_2_ production rate (*V̇*_CO_2__) were calculated in ml min^−1^ according to Lighton (2019):
(1)

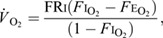

(2)

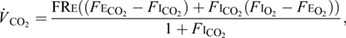
where FRi is the incurrent dry flow rate, *F*i_O_2__ and *F*i_CO_2__ are the incurrent fractions of oxygen and carbon dioxide, and *F*e_O_2__ and *F*e_CO_2__ are the excurrent fractions. The airstream was chemically scrubbed of water vapour and carbon dioxide before measuring O_2_ [using Drierite and Ascarite(II); see above]. We mathematically scrubbed water vapour before calculating *V̇*_CO_2__ following equation 8.7 in Lighton (2019). We converted oxygen consumption to RMR (W) according to Lighton et al. (1987):
(3)

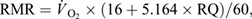
where RQ is the respiratory quotient (RQ=*V̇*_CO_2__/*V̇*_O_2__).

### Body temperature data calibration

We calibrated thermocouples and PIT tags in the climate chamber (which had been factory calibrated shortly before the start of the experiment) at 35, 40 and 45°C, a range of temperatures that span daily variation in body temperature in this study species. A calibration unit consisted of a thermocouple with a PIT tag attached close to the thermosensitive junction. This setup was replicated 2–3 times per antenna, yielding a total of 33 comparisons between thermocouple/PIT tag and climate chamber temperature. We used linear regressions based on these relationships to calibrate each body temperature measurement method.

### Sample size

Of the 76 great tits that were initially included in the experiment, four (SC *n*=2; CL *n*=1; *n*=1 not yet assigned to a treatment), died before (3) or during (1) respirometry and were not included in any analyses. Of the 72 remaining birds, one died on the day after (CL) respirometry, and another bird had to be euthanized because of declining health after the experiment (also CL). Neither of these birds showed any deviations during respirometry and they were, thus, included in the analyses of metabolic rate and body temperature (see Fig. S2 for body temperature traces from these birds, 2KX08963 and 2KX08975). Four birds (CU *n*=2, CL *n*=2) lost their thermocouple during respirometry and were excluded from the dataset. Furthermore, one bird (2KV07902, CL–see temperature profiles in the Fig. S2) lost the thermocouple early during the night and we were only able to secure body temperature and metabolic measurements for one ambient temperature (25°C). As the method itself was lost when the thermocouple was dislodged, we excluded all further metabolic rate measurements for this individual. Finally, as we set the thermocouples to only record temperatures between 30 and 50°C (a lower range of temperatures improves accuracy during digitalization of the analog voltage signal), we lost temperature data for one bird (2KX08973) for parts of the night when skin temperature dropped below 30°C (see Fig. S2). As the thermocouple was always attached to the bird, metabolic rate data from this individual were included for all ambient temperatures. Single erratically occurring IP or SC values >45°C in data series of normal night-time body temperatures (i.e. 35–40°C) were removed from the temperature profiles (41 observations of 2,296,539 total; from 2 CU, 6 SC, 1 IP birds) as they were obvious mis-readings. Thus, the final dataset, used in analyses of metabolic rate and body temperature, consisted of 68 birds (Table S1) that provided data for all ambient temperatures (66 birds) or for at least one of the ambient temperatures (2 birds). Body mass did not differ between methods (Table S1), either at capture (ANOVA: *F*_4,63_=0.77, *P*=0.55) or before (ANOVA: *F*_4,63_=0.47, *P*=0.76) or after the measurements (ANOVA: *F*_4,63_=0.37, *P*=0.83) and group composition in terms of age and sex did not differ between methods (Pearson's chi-squared test: age χ^2^_4_=0.05, *P*=1; sex χ^2^_4_=1.20, *P*=0.88).

### Data extraction

Oxygen consumption was calculated as the mean value of the most stable 2 min period per measurement cycle (Fig. S1A) using Expedata software, and RMR for each ambient temperature was defined as the lowest of those periods. Mean body temperature was then extracted for time periods corresponding to the start and end of the designated RMR period. Because of interference/signal strength issues, one bird (IP) lacked time-matched body temperature recordings in two ambient temperatures. For this bird, we defined body temperature as the single measurement closest in time to the RMR period (−6 min and −5 s, respectively).

### Statistical analyses

All models were performed in R, version 4.2.0 (http://www.R-project.org/). We used linear mixed models (*nlme* package, Pinheiro and Bates, 2000) to evaluate the effect of method, ambient temperature, age and body mass on body temperature and metabolic rate. Method, ambient temperature and age were treated as factors while body mass was added as a covariate. Age was included in the models as we know from previous studies that age of the bird may influence thermoregulation and energy expenditure (Andreasson et al., 2020a). Individual bird ID was added as a random factor in both models. We also allowed for method to interact with ambient temperature and age, respectively, in both models. Our initial candidate set of models were fitted with maximum likelihood (ML) and we selected final models by comparing goodness of fit for models with and without interactions based on Akaike's information criterion for small sample size (AICc) (Akaike, 1973). For metabolic rate, the model without interactions performed best (ΔAICc≥8.6) compared with all other models, and for body temperature, the model with an interaction between method and ambient temperature performed best (ΔAICc≥2.8) compared with all other models. All original main effects were retained. The two final models selected were then fitted using restricted maximum likelihood (REML) to obtain parameter estimates. Mixed models were fitted allowing for method-specific variances (*varIdent* in *nlme* package). Statistics for fixed effects were calculated using Wald chi-square tests (*Anova* in *car* package; Fox and Weisberg, 2019) and the random effect of bird ID was evaluated using likelihood ratio tests. Model estimates are estimated marginal means (*emmeans* package; https://CRAN.R-project.org/package=emmeans) and all pairwise comparisons (*emmeans* package) were Bonferroni corrected for multiple testing.

To estimate temporal variability of individual temperature profiles (see Fig. S2), we calculated a coefficient of variation (CV) for each individual, and also estimated a consecutive disparity index (*D*) for each individual (following Fernández-Martínez et al., 2018). The disparity index was calculated by summing all the natural logarithms of the proportion of consecutive body temperature values and dividing by the number of values minus one (equation 3 in Fernández-Martínez et al., 2018). Thus, *D* has the advantage of being independent of mean temperature of the time series but also of taking the chronological order of measurements into account, which CV does not. All body temperature measurements from both thermocouples and implanted tags were rounded to one decimal place (resolution of the BioTherm 13 PIT-tag) and binned into 1 min means (to facilitate comparison between thermocouples and tags) before CV and *D* were calculated. We used Welch's ANOVA (allowing for unequal variances between methods) to determine whether variability according to CV or *D* was different between the body temperature measurement methods, and pairwise *post hoc* comparisons were made using the Games–Howell test (*rstatix* package; https://CRAN.R-project.org/package=rstatix).

## RESULTS AND DISCUSSION

**Fig. 2.**
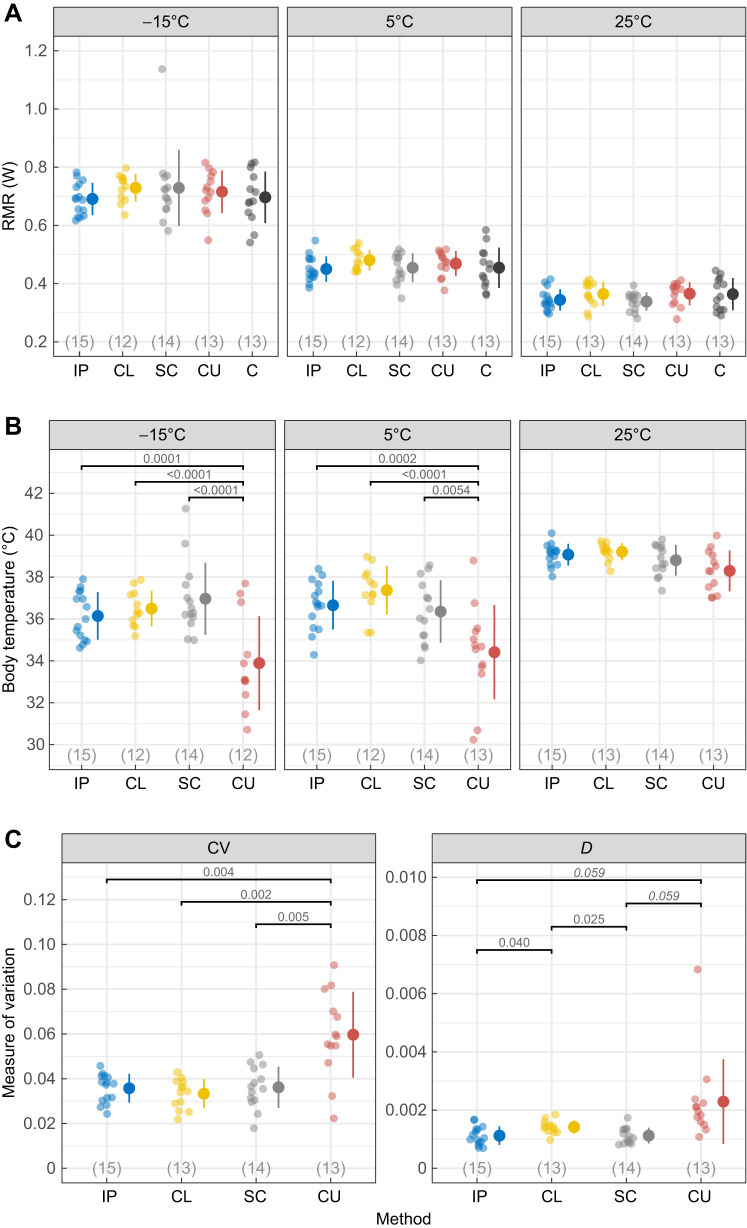
**Resting metabolic rate and body temperature of wild-caught great tits (*Parus major*) using the four methods.** (A) Resting metabolic rate (RMR) and (B) body temperature at three ambient temperatures (−15, 5 and 25°C). (C) Variability, measured as the coefficient of variation (CV) and as the consecutive disparity index (*D*) for body temperature profiles. Body temperature was measured by an intraperitoneal implant (IP), a cloacal thermocouple (CL), a subcutaneous implant (SC) or a cutaneous thermocouple (CU). An un-instrumented control group (C) was also included for RMR measurements in A. Circles and error bars show group mean values (±1 s.d.) and semi-transparent circles show the raw data. Significant pairwise differences between methods are indicated by bracketing rules with corresponding *P*-values (italicized when 0.05<*P*<0.1). Sample sizes per method and temperature are reported in each panel.

There was no effect of method on RMR at any experimental temperature (Table 1, Fig. 2). RMR increased with decreasing ambient temperature so that birds increased RMR by 29% at 5°C compared with that at 25°C, and by 53% at −15°C compared with that at 5°C (Table 1, Fig. 2). RMR increased with body mass (0.026 W g^−1^, *P*<0.0001), but did not differ between the two age categories (*P*=0.30; Table 1). Thus, RMR was never affected by how body temperature was measured. In addition, none of the methods increased RMR above that of un-instrumented, control birds (Fig. 2). This does not imply that the methods are non-invasive and perceived as non-stressful, but merely that body temperature measurement using a range of common methods does not cause an additive stress-induced increase in metabolic rate on top of that imposed by respirometry itself. Previous studies have shown that repeated cross-sectional sampling of cloacal or rectal temperature can be stressful, reflected as an increase in body temperature and heart rate (a common proxy for metabolic rate; reviewed in Green, 2011), in both birds (Cabanac and Aizawa, 2000; Cabanac and Guillemette, 2001) and mammals (Bouwknecht et al., 2007). In contrast, we found no support for stress-induced alterations to either tissue temperature or metabolic rate when great tits were instrumented with a permanently mounted cloacal thermocouple. Nor did we find evidence suggesting that tethering to a thermocouple alone (i.e. without insertion; in the CU group) was associated with stress-induced changes to temperature or RMR. Thus, any additional stress imposed by continuous thermocouple measurements was either too transient to be registered by our whole-night recordings or did not add to any potential stress from handling and respirometry. This difference might be explained by the use of repeated, single-point measurements of cloacal or rectal temperature in the studies above, which confounds stressors from thermocouple insertion and handling. Future studies could benefit from measuring other stress markers, such as circulating glucocorticoids (reviewed in Dantzer et al., 2014), or other downstream measures such as glucose or free fatty acids (reviewed in Breuner et al., 2013), to improve our understanding of the perceived invasiveness of different measurement techniques.

**Table 1.**
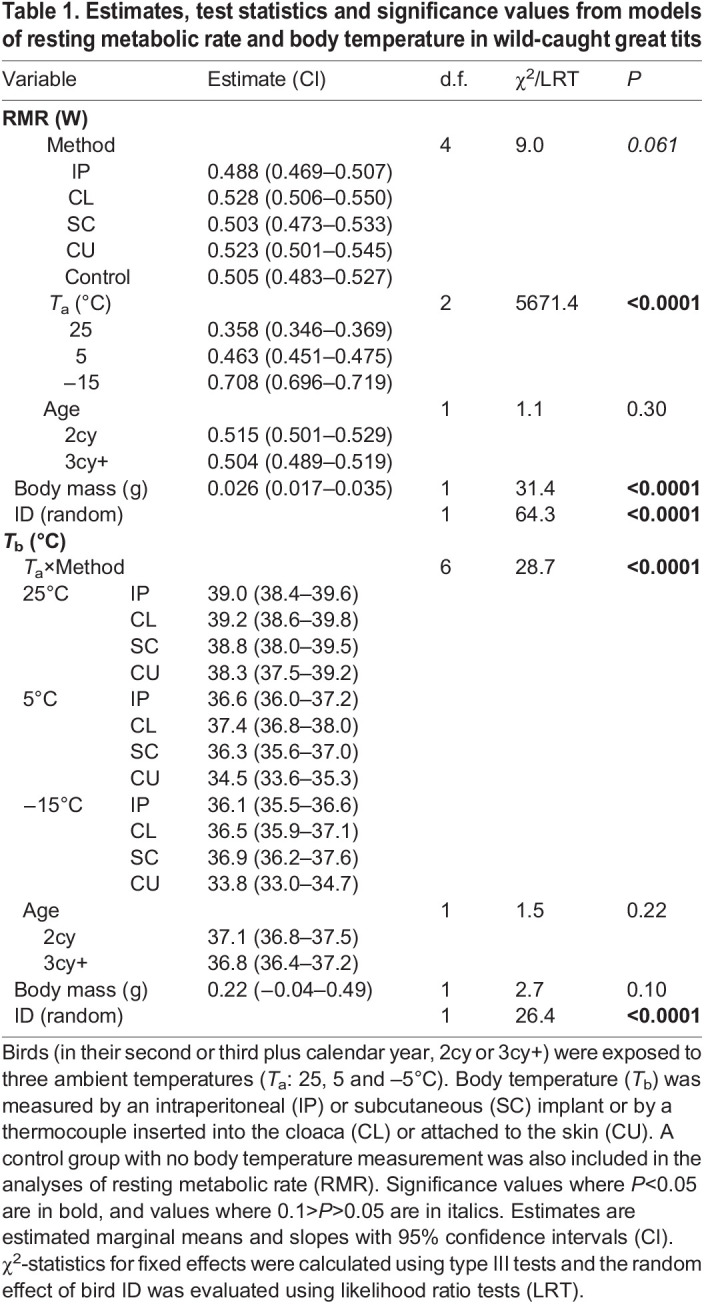
Estimates, test statistics and significance values from models of resting metabolic rate and body temperature in wild-caught great tits

The difference in body temperature between measurement methods was dependent on ambient temperature (*P*<0.0001; Table 1, Fig. 2). Accordingly, body temperature did not differ between methods at an ambient temperature of 25°C (all pairwise comparisons *P*≥0.56), but at both 5°C and −15°C, CU birds had significantly lower body temperature compared with that for all other methods (by 1.8–2.9 and 2.3–3.1°C, respectively; Table 1, Fig. 2). When averaging across methods, body temperature was lower at 5°C and −15°C compared with that at 25°C (pairwise comparisons, *P*<0.0001) but did not differ between 5°C and −15°C (pairwise comparison, *P*=0.16). Neither age of the bird (*P*=0.22) nor body mass (*P*=0.10) affected body temperature (Table 1). Hence, we found partial support for the prediction that any differences between internal and peripheral body temperature measurement methods would be the most pronounced at low ambient temperatures. Previous studies have shown that, in smaller animals, body temperature under or close to the skin may correlate well with core temperature (Brown and Bernard, 1991; Dausmann, 2005; Wacker et al., 2012; Nord et al., 2013; Oswald et al., 2018). However, this correlation is probably affected both by the direct effect of ambient temperature, which has a disproportionate effect on peripheral sensors (e.g. a larger thermal gradient between skin and core in colder ambient temperature; see Audet and Thomas, 1996; Barclay et al., 1996; Willis and Brigham, 2003; Wacker et al., 2012; Andreasson et al., 2020a; but see Oswald et al., 2018), and by changes in vasomotor state triggered by variation in ambient temperature. Body size will also affect the thermal gradient between core and shell, whereby larger animals may maintain a substantially larger temperature gradient between the core and surface (e.g. Mole et al., 2016) and so can control heat loss via regulation of skin temperature better compared with smaller animals (Phillips and Heath, 1995).

Individual body temperature profiles in the CU group were 65–79% more variable compared with all other methods, when expressed as temporal variability (i.e. CV; *F*_3,26.8_=7.0, *P*=0.0013). However, when taking the chronological order of body temperature measurements into account, there was an overall difference in variability between methods (i.e. *D*; *F*_3,27.0_=6.1, *P*=0.0027) (Fig. 2) but CU profiles only tended to be more variable compared with those for SC and IP (*P*=0.059 and *P*=0.059, respectively) and there was no difference between CU and CL (*P*=0.19). CL profiles were 27% more variable compared with both SC and IP profiles (*P*=0.025 and *P*=0.040, respectively). Taking these two indices of variability into account, our interpretation is that cutaneous measurements were more variable overall, but that there are substantial differences in variability between individuals and that both methods that use thermocouples (CL and CU) are, or tend to be, more variable compared with implanted sensors (Fig. 2). For cloacal thermocouples (CL), this increased variability was only visible when taking the chronological order of measurements into account.

Overall, the usefulness and applicability of cutaneous measurement might be limited, at least in the settings assessed here. However, the fact that we found no difference in body temperature between the IP and SC groups suggests that peripheral temperature measurement can still be a useful predictor of core body temperature in this model even in ambient temperatures far below thermoneutrality, provided the instrument is placed subcutaneously. Oswald et al. (2018) reached a similar conclusion when measuring a similarly sized bird species under warm conditions. Thus, peripheral measurements should not be treated as one and the same as they may provide considerably different estimates depending on whether the sensor is glued onto the skin or implanted below it. We stress that the results presented here apply to small birds at thermoneutrality and below, and it would be relevant to test whether these patterns are consistent across other study systems where organisms differ in size and degree of homeothermy.

We have shown that body temperature measurements in great tits, irrespective of method, do not increase RMR across a range of ambient temperatures spanning thermoneutrality to far below it. In the cross-sectional dataset, body temperature estimates also did not differ between methods, except for cutaneous measurements, which were lower and more variable below thermoneutrality. Future studies should test whether this pattern is consistent across other study systems. However, our results add flexibility for researchers measuring body temperature in similar, small bird models and should be reassuring for those that aim to characterize thermoregulatory phenotypes by simultaneous measurement of body temperature and metabolic rate in such systems.

## Supplementary Material

10.1242/jexbio.246321_sup1 Supplementary information
