# Supplementary material for: Measuring body temperature in birds – the effects of sensor type and placement on estimated temperature and metabolic rate
Source: J Exp Biol. 2023 Dec 12;226(24):jeb246321. doi: 10.1242/jeb.246321 (PMC10753514; doi:10.1242/jeb.246321)
Supplement: Supplementary information [file jexbio-226-246321-s1.pdf]

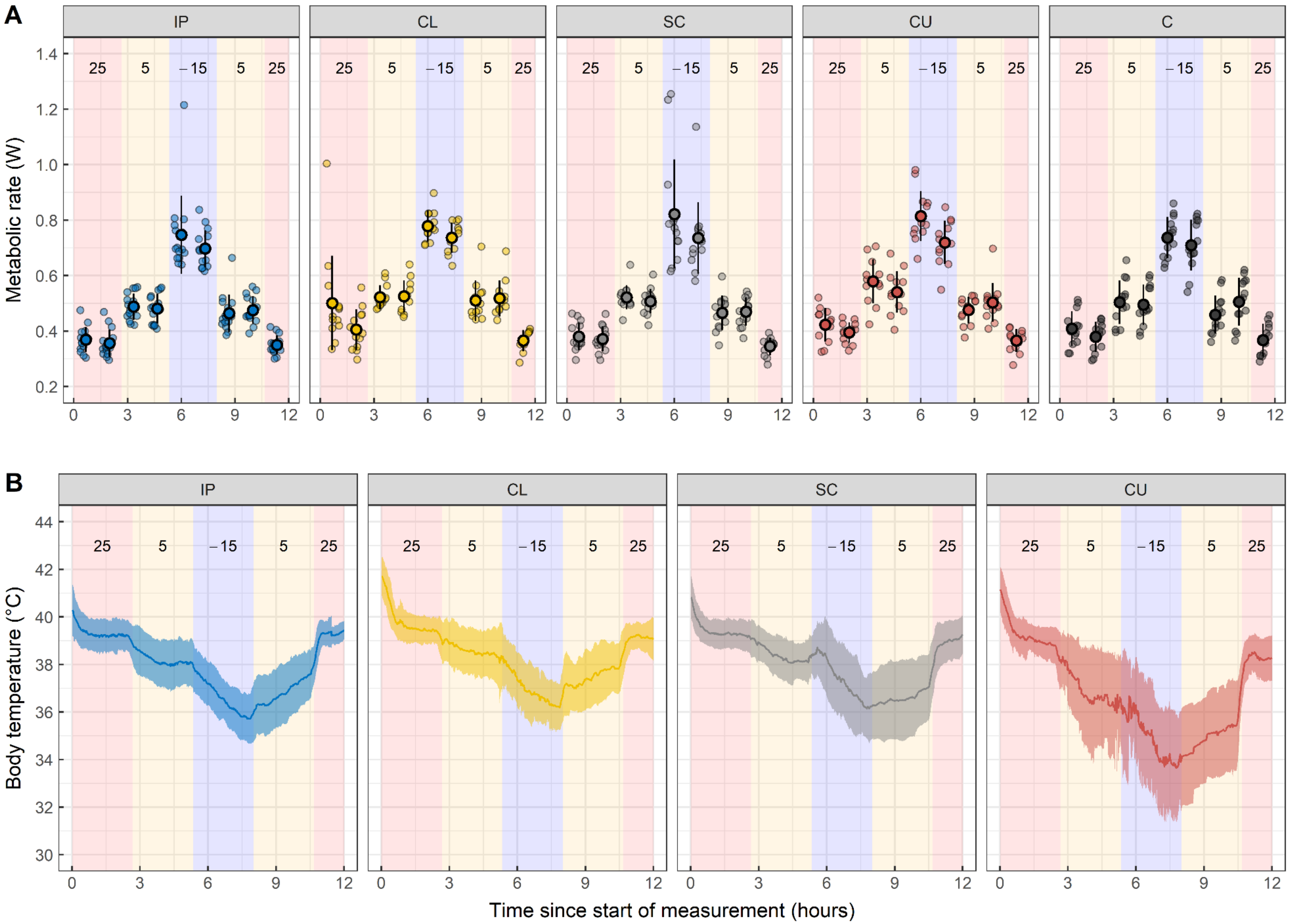

Journal of Experimental Biology • Supplementary information

Fig. S1. A) Metabolic rate profiles of wild-caught great tits (*Parus major*) during nine measurement cycles spanning sunset to sunrise in three different ambient temperatures; and B) mean (±1 s.d.) body temperature profiles based on all birds that were included in the analyses of body temperature We recorded body temperature continuously throughout the night using one of four methods: an intraperitoneal implant (IP), a cloacal thermocouple (CL), a subcutaneous implant (SC) or a cutaneous thermocouple (CU). An un-instrumented control group (C) was also included in the experiment. In A), individual data are plotted using semi-transparent plotting symbols whereas solid points and error bars show mean metabolic rate (±1 s.d.).

Journal of Experimental Biology • Supplementary information

A

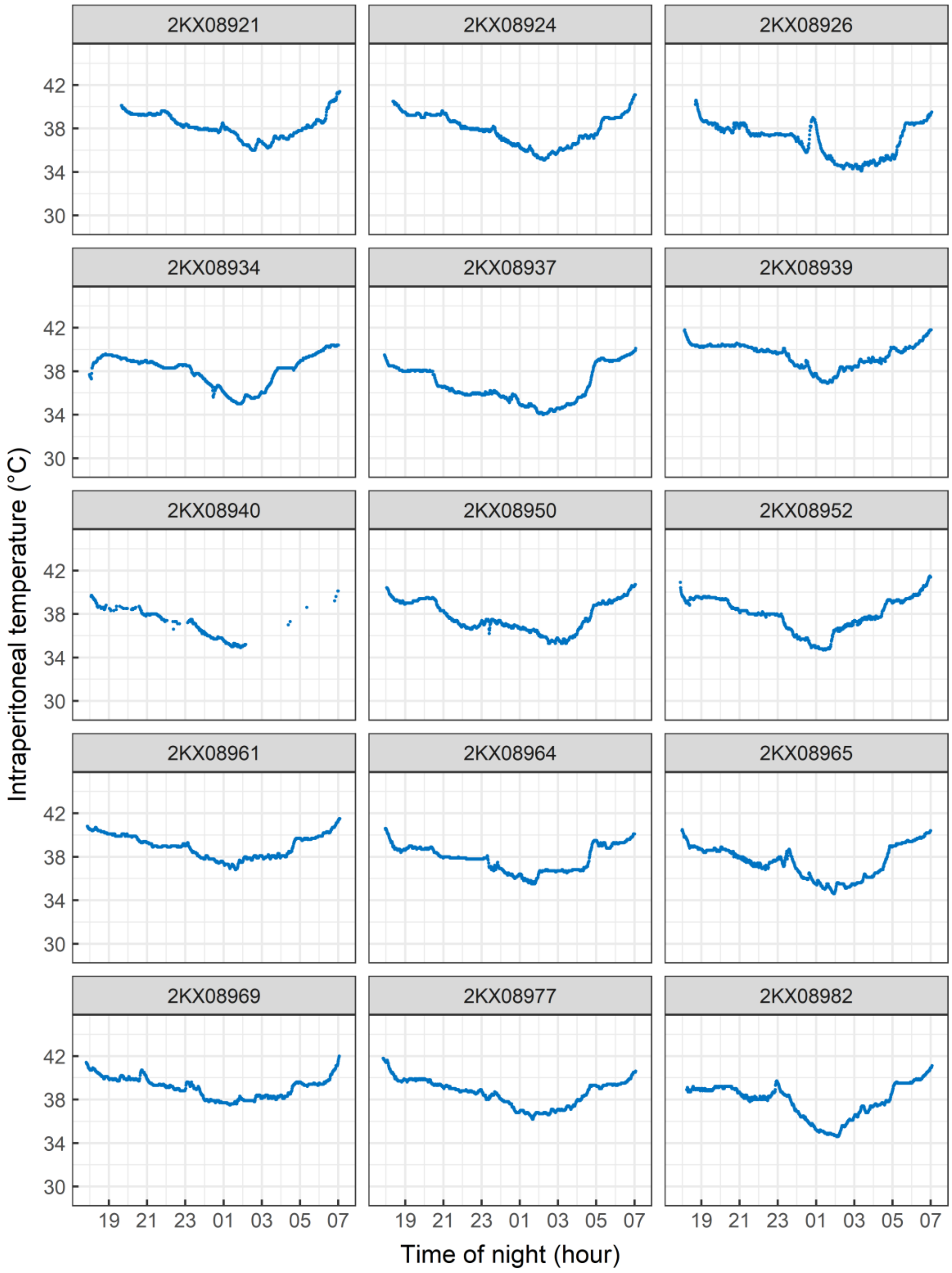

Journal of Experimental Biology • Supplementary information

B

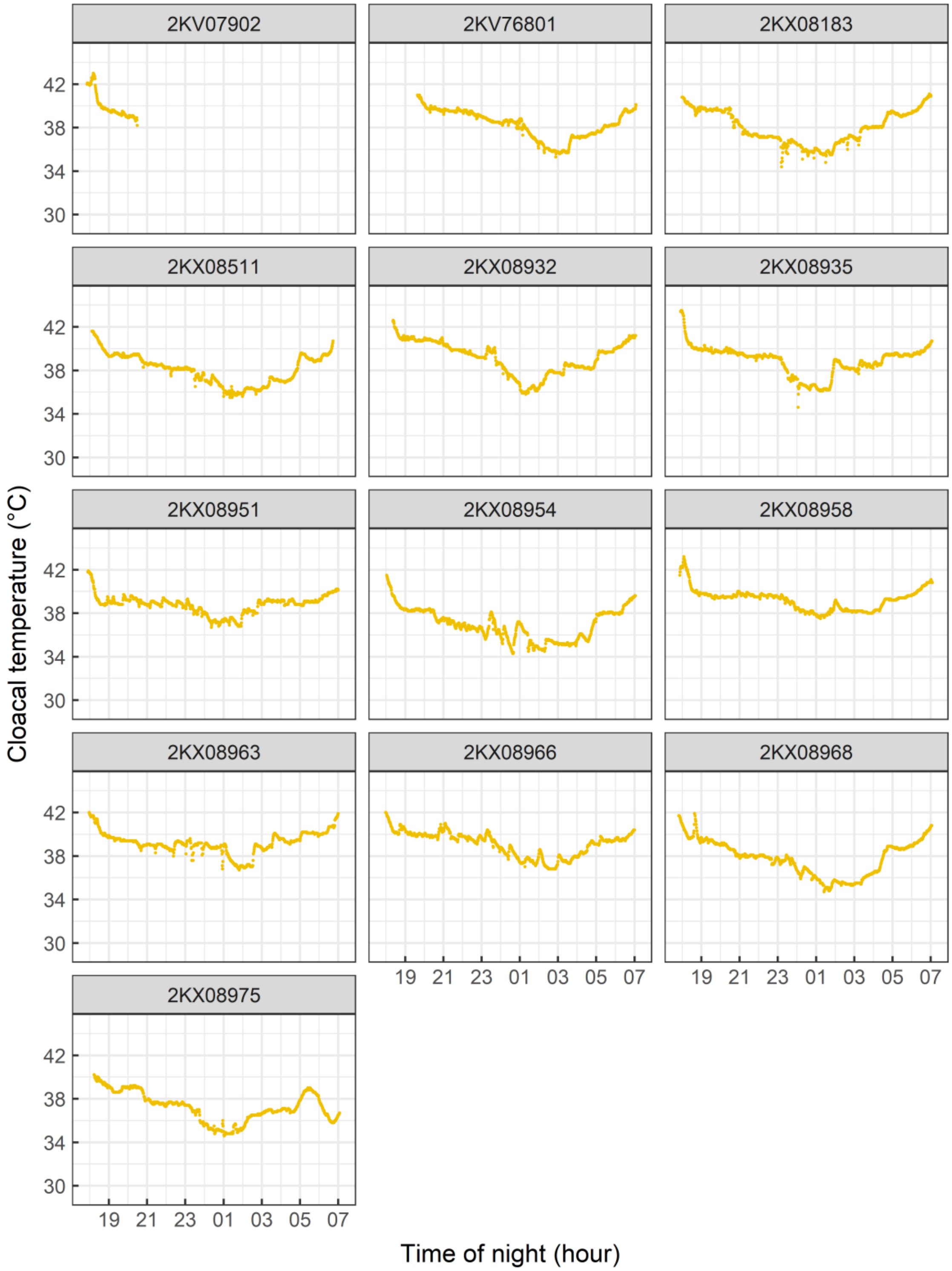

Journal of Experimental Biology • Supplementary information

C

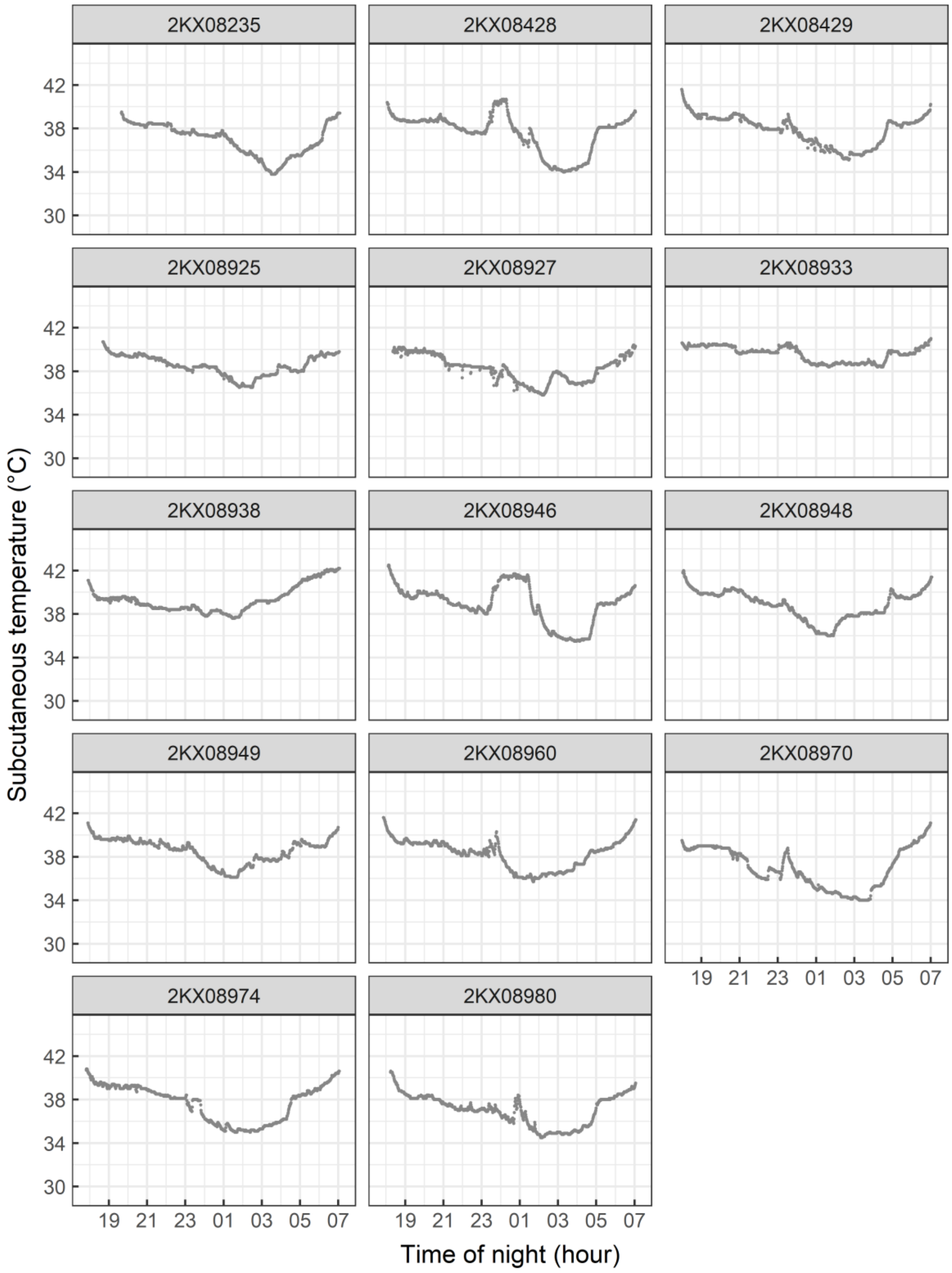

Journal of Experimental Biology • Supplementary information

D

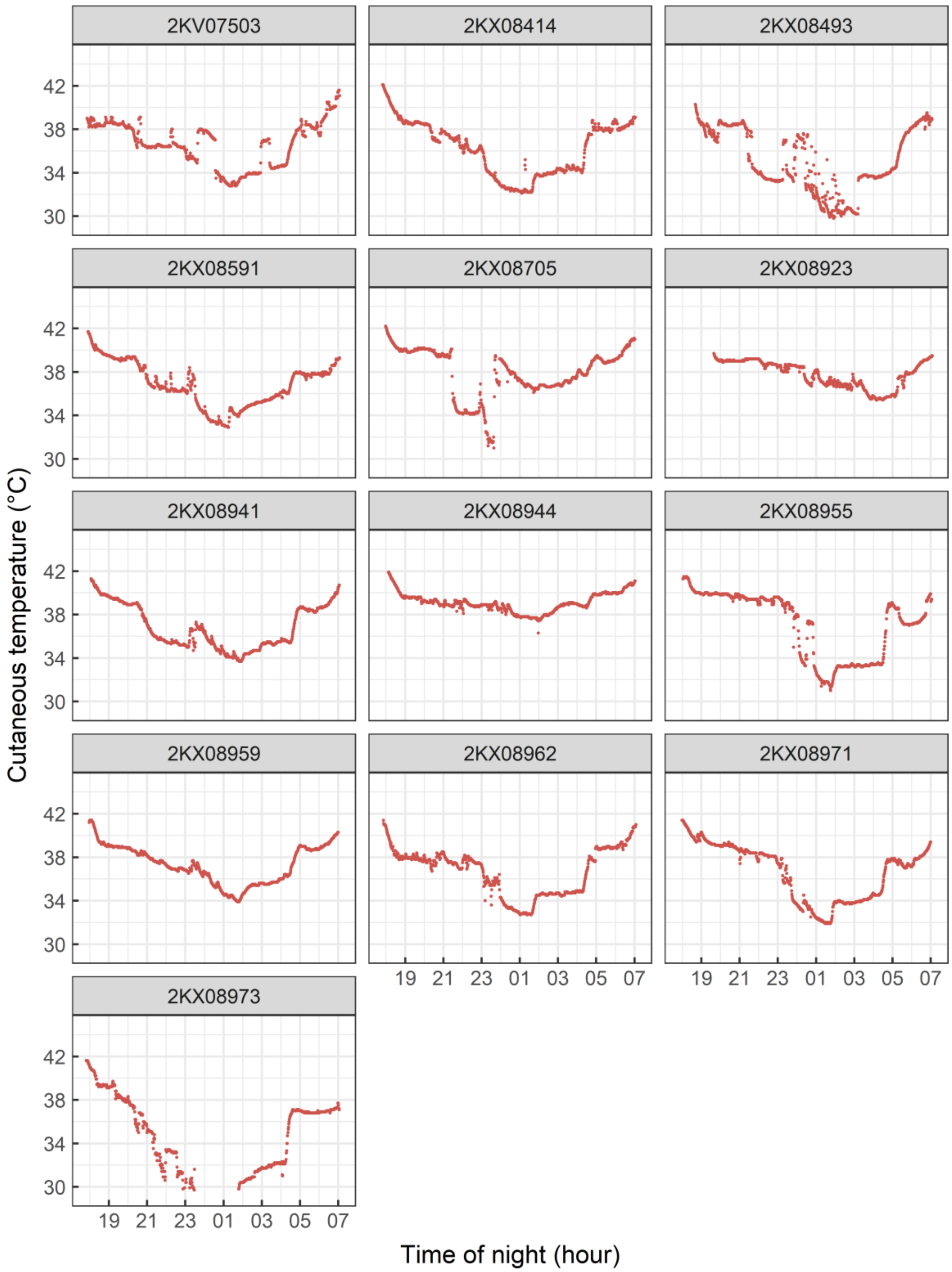

Journal of Experimental Biology • Supplementary information

Fig. S2. Body temperature profiles from sunset to sunrise for all great tits (*Parus major*) included in the analyses. Panel groups represent body temperatures measured using: A) an intraperitoneal implant - IP, B) a cloacal thermocouple - CL, C) a subcutaneous implant - SC, and D) a cutaneous thermocouple - CU, as detailed in the main text. The thermocouple did not record below 30 °C and, thus, values ≤ 30°C are not included in temperature profiles. Values above 45 °C were also removed from the data set since they were always the result of technical artefacts (41 observations of 2 296 539 total; from 2 CU, 6 SC, 1 IP birds). For two birds (CL: 2KV07902 and 2KX08511), we manually excluded data recorded after the birds had dislodged the thermocouple.

Table S1. Initial and final sample sizes for all methods of body temperature measurement, split across ages and sexes, and body mass (± 1 s.d.) at ringing, and before and after the measurements. Biometric data are based on the final sample sizes.

		Experimental method category				
		**IP**	**CL**	**SC**	**CU**	**C**
Initial sample size		15	16	16	15	13
Final sample size		15	13	14	13	13
Age	2cy	8	7	8	7	7
	3cy+	7	6	6	6	6
Sex	Female	7	5	8	7	7
	Male	8	8	6	6	6
Body mass - ringing (g)		18.96±1.02	18.88±0.79	18.49±1.22	18.38±1.14	18.88±1.29
Body mass - before measurements (g)		18.01±1.18	17.65±0.92	17.91±1.09	17.48±0.97	17.75±1.43
Body mass - after measurements (g)		16.46±1.32	16.20±1.16	16.32±0.95	15.95±0.96	16.15±1.42

Journal of Experimental Biology • Supplementary information
